# Falling Short: The Contribution of Central Insulin Receptors to Gait Dysregulation in Brain Aging

**DOI:** 10.3390/biomedicines10081923

**Published:** 2022-08-09

**Authors:** Sami L. Case, Hilaree N. Frazier, Katie L. Anderson, Ruei-Lung Lin, Olivier Thibault

**Affiliations:** Department of Pharmacology and Nutritional Sciences, University of Kentucky College of Medicine, 780 Rose Street, Lexington, KY 40536, USA

**Keywords:** insulin resistance, gerontology, ambulatory function, signaling

## Abstract

Insulin resistance, which manifests as a reduction of insulin receptor signaling, is known to correlate with pathological changes in peripheral tissues as well as in the brain. Central insulin resistance has been associated with impaired cognitive performance, decreased neuronal health, and reduced brain metabolism; however, the mechanisms underlying central insulin resistance and its impact on brain regions outside of those associated with cognition remain unclear. Falls are a leading cause of both fatal and non-fatal injuries in the older population. Despite this, there is a paucity of work focused on age-dependent alterations in brain regions associated with ambulatory control or potential therapeutic approaches to target these processes. Here, we discuss age-dependent alterations in central modalities that may contribute to gait dysregulation, summarize current data supporting the role of insulin signaling in the brain, and highlight key findings that suggest insulin receptor sensitivity may be preserved in the aged brain. Finally, we present novel results showing that administration of insulin to the somatosensory cortex of aged animals can alter neuronal communication, cerebral blood flow, and the motivation to ambulate, emphasizing the need for further investigations of intranasal insulin as a clinical management strategy in the older population.

## 1. Introduction

In the United States, falls are a leading cause of both fatal and nonfatal injuries in older adults [1]. On average, 30–40% of people over the age of 65 and 50% of people over the age of 80 will experience a fall each year [1,2,3,4]. Many of these events are associated with injury, with one study reporting that in women >70 years old, 41% of falls resulted in minor injuries while 6% resulted in major injuries (i.e., head trauma, fractures, or lacerations) [2,5,6]. Additionally, falls account for >60% of emergency room visits in patients who are 65 or older [5], of which 5% conclude with hospitalization [6]. The likelihood of incurring a fall-related injury depends on a variety of factors, such as the height and velocity of the event [7,8] and the overall health of the individual [9,10]. Interestingly, sex also appears to play a role [11], as older women are less likely to experience a fall [12,13] but more likely to sustain an injury (either minor or major) compared to older men [1]. In addition to elevating the risk of short-term injury, falls in older adults also lead to increased risk of morbidity, chronic medical complications, and admission into long-term care facilities [14,15,16,17]. While death from falls is much less common, long-term complications associated with fall events are still a significant contributor to mortality in older populations [18]. In fact, approximately 2% of injurious falls in older adults result in death [19], and this statistic increases with age. Further, fear and anxiety surrounding the outcome of these events seems to be more prevalent in this particular population, with one study reporting that 80% of older women would prefer death over an injurious fall and placement into a long-term care facility [20].

While the increase in falls with age is undisputed, central mechanisms responsible for this have not been investigated thoroughly in animal models of aging. Recently, however, it has become clear that chronic conditions, including stroke, diabetes and dementia are intrinsic risk factors that are not only critical when considering the etiology of falls with aging, but importantly, may also represent therapeutic targets for reducing fall risk with age. Evidence from a recent meta-analysis investigating over 14,000 patients identified a significant increase in fall risk in patients with diabetes compared with healthy subjects [21], further supporting prior evidence from the Longitudinal Ageing Study, that individuals with diabetes exhibit more frequent falls compared to healthy adults [22]. The links between peripheral metabolic dysregulation and falls have been reviewed recently with careful attention to the potential involvement of sensorimotor dysregulation, musculoskeletal dysfunction, and pharmacological complication. It seems clear that repeated hypoglycemic events in patients with diabetes likely contribute to an increased risk of falls [23]. More recently, polypharmacy use has been shown to weaken glycemic control, giving rise to increases in dizziness and falls [24]. 

Clearly, characterizing the pathological changes and mechanisms that underlie age-dependent gait impairments, including processes associated with metabolic dysfunction, is a worthwhile endeavor, as these impairments significantly increase the risk of falling in the older population. However, despite enormous advances in the identification of peripheral mechanisms that contribute to altered ambulatory function and increased fall risk in older individuals, there is still a paucity of information highlighting the potential *central* components of ambulatory distress with age. Additionally, there is currently a lack of effective therapeutic treatments designed to target these central processes. Here, we discuss alterations in central modalities that may contribute to gait dysregulation with age that are sensitive to insulin in the primary somatosensory cortex, and which clearly participate in locomotor activity. We wish to raise awareness that these central functions that control ambulation are a valuable therapeutic target for the prevention of falls with age.

## 2. Current Therapeutic Approaches

While it is undeniable that injurious falls significantly impact the quality of life of those affected, these events also exert an immense financial burden on society. In fact, as of 2015, the estimated cost of care associated with fall injuries in individuals ≥ 65 years old was nearly $50 billion in the US [25]. The use of exercise interventions such as physical therapy, yoga, and Tai Chi all appear to improve ambulatory performance in the older adult [26,27,28], yet lack of accessibility to these programs as well as low patient compliance limits their potential benefit. Additionally, there are currently very few effective therapeutic approaches available to address fall risk in these individuals beyond recommending nutritional changes and maintenance of optimal vitamin D status [29,30,31,32,33,34]. While rivastigmine has been shown to reduce fall frequency by 45% in patients diagnosed with Parkinson’s disease (PD), these results did not identify potential pathways or cellular targets mediating this effect [35], and it appears that other cholinomimetics do not reliably improve imbalance-related falls [36,37]. Recently, the use of fampridine, a potassium channel blocker, in individuals with multiple sclerosis appeared to be beneficial in improving gait speed [38], while memantine, an anti-dementia drug, was able to improve gait variability in patients diagnosed with Alzheimer’s disease (AD) [36]. However, it is not clear if any of these drugs could be beneficial to the normal aging population, nor what central modalities they target. Finally, it is important to note that the use of certain prescription medications (e.g., benzodiazepines, α- and β-blockers) appears to correlate with, and often exacerbate, the fall risk in individuals >70 years old [39,40]. This produces a significant complication with effectively treating falls, as these medications are often prescribed for other age-associated dysregulations such as insomnia, high blood pressure, and anxiety. Because of this, attempting to identify new therapeutic avenues that more directly target the physiological processes underlying these events without negatively impacting other factors of daily life is highly relevant.

## 3. Contribution of Peripheral Systems and Special Senses to Gait Dysregulation

While there has been a large amount of work focused on extrinsic physical interactions that can be modified to reduce fall risk in the older adult (i.e., type of shoes, mobility aids, environmental changes, etc.), even more intrinsic factors have been identified; these include peripheral alterations such as sarcopenia and metabolic disorders as well as impairments in special senses, such as vestibular and visual systems, which ultimately reduce activities of daily living.

### 3.1. Peripheral Systems

In cases where physical rehabilitation and balance training are used to improve gait dysregulation in older individuals, it is not clear which physiological changes underlie the benefits of these therapies. This is further complicated by the presence of comorbidities associated with aging. For example, age-dependent musculoskeletal alterations have been thoroughly implicated as a contributing factor to frailty and increased risk of falls [41]. Lower limb weakness resulting from sarcopenia (the deterioration of muscle tissue with age) has also been shown to impede the ability to stand, reduce gait speed, and impair balance [42]. Further, the link between the increased prevalence of falls and sarcopenia in the older adult is clear, and this underlies many validated approaches for the management of falls in this population, particularly the strengthening of peripheral muscles using physical training and exercises such as Tai Chi [43,44] and yoga [26,44]. However, in addition to limiting lower limb function, sarcopenia is also associated with an elevated risk of developing metabolic dysregulation [42], likely due to decreased peripheral glucose uptake resulting from diminished muscle mass. Interestingly, gait impairments are intensified by peripheral metabolic disorders such as Type-2 diabetes mellitus (T2DM) [45], particularly in older women [46,47]. Studies in older adults have shown that T2DM is also associated with an increased fear of falling and lower balance confidence [45,48,49]. Similarly, one meta-analysis of over 13,000 patients indicated that those with obesity and diabetes had a higher risk of falling and worsened recovery outcomes compared to healthy individuals [50], while another analysis of over 1 million patients reported that obesity increased the likelihood of multiple falls in individuals over the age of 60 [48]. While much has been learned regarding the role of physical exercise in treating these peripheral alterations, other intrinsic factors, particularly changes in vestibular and visual senses, have also been implicated in mediating poor stability outcomes with aging.

### 3.2. Special Senses

In addition to peripheral dysregulations, special sense impairments, such as vestibular and vision loss, are also associated with falls and poor gait in older individuals [49,51]. Vestibular signals contribute to balance and walking, where the otolith organs and semicircular canal (SCC) output converge to guide the control of balance and posture during ambulation. In the older adult, decreases in SCC function result in longer stride length and stance time, in addition to slower cadence [52]. While vestibular hypofunction in advanced ages can lead to dizziness, postural instability, and unsteady gait [53,54], previous studies show that over 30% of people living at home and over 50% in assisted living facilities experience at least one fall per year, without experiencing dizziness [55,56,57,58], suggesting only a partial contribution of vestibular function to gait dysregulation with aging. 

The vestibular system communicates regularly with the visual system to maintain stability through reflexes, such as the vestibulo-ocular reflex, which helps to stabilize gaze. Much research has also investigated the impact of vision loss in age-dependent gait alterations, as it plays a large role in coordination and planning of movement in addition to balance. One study recently showed that patients with age-related macular degeneration had significantly slower walking speeds and stride velocities [59], while another reported that reduced contrast sensitivity, but not visual acuity, with age is associated with decreased stride lengths [60]. Further, slower gait and cadence, shortened stride length, and lengthened double support time are all exacerbated in extreme or changing lighting conditions, and is not shown to be dependent on fear of falling [61]. 

**Figure 1:**
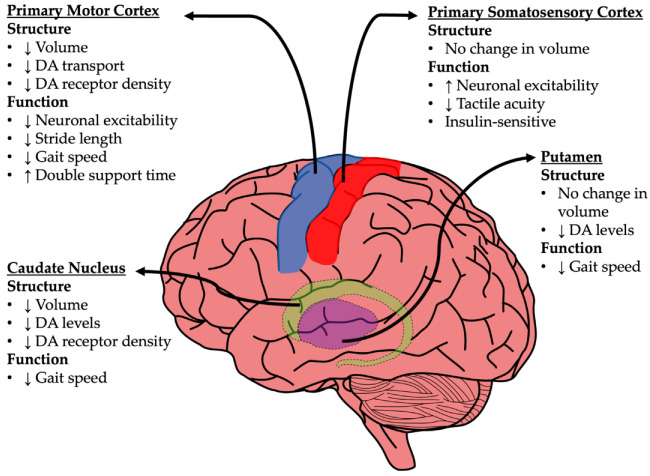
**Aging-related structural and functional changes in key brain regions that control gait**.

Currently, a large amount of work has highlighted several central and peripheral intrinsic factors associated with comorbidities of aging that are tied to ambulatory distress, including hypertension, muscle weakness and fatigue, poor visual acuity, loss of vestibular function, weak tendons and/or joints, and reduced sensory modalities. However, while this work has given rise to a rich body of associative clinical studies, few, if any, have directly investigated the role of less-characterized brain regions that are associated with motor and sensory ambulatory control, such as the basal ganglia or the primary motor and somatosensory cortices (see Figure 1).

## 4. Neuroanatomical Changes in Gait Processing Centers

The field of brain aging has often focused on cognitive- and memory-associated functions, and most investigations were conducted in the hippocampus and associated cortices [62]. However, accumulating evidence shows that superficial layers in the primary somatosensory cortex receive inputs from the thalamus and cortical areas [63,64,65] associated with limb movement and sensory encoding [66]. Other critical regions include the dorsal basal ganglia and the motor thalamus [67,68]. Given that age is positively correlated with the number of falls an individual experiences [69,70,71], that dysregulation in hippocampal, cortical, and thalamic pathways (all of which are part of the gait processing network) predicts cognitive decline in AD [72,73], and that cognitive status and falls share common mechanisms, it becomes difficult to ignore central aspects of motor/sensory function in aging.

### 4.1. Basal Ganglia

The basal ganglia, including the caudate nucleus and putamen, are heavily involved in the tuning of voluntary motor output from the motor cortex. Specifically, these regions act in unison to determine the most appropriate motor behavior, including learned behavior (i.e., walking, running, etc.). Interestingly, in these areas, four decades of research have shown age-dependent reductions in dopamine levels as well as reduced D1 and D2 receptor expression [74,75,76,77,78]. However, while changes in dopaminergic signaling in the basal ganglia with age have been well-described, few studies have investigated the role of these biomolecular changes on gait function. In older subjects, smaller caudate nucleus volumes have been correlated with slower walking speed [79]. Additionally, age-related neurological diseases that are associated with altered ambulatory function, such as AD, have also been tied to dysregulations in the basal ganglia, where changes in gait speed are associated with AD pathology, particularly in the posterior putamen [80]. Still, while it is well-known that the basal ganglia contribute to the tuning of motor behavior and that aging can alter basal ganglia function, their impact on gait in the context of normal aging is not well characterized, and it is clear that other neuroanatomical gait centers are at play [81,82].

### 4.2. Primary Motor Cortex (M1)

The M1 is responsible for the execution of movement and is the last cortical area involved in the process of motor output. This area sends long projections to lower CNS structures, including lower motor neurons in the spinal cord that control leg movements. As with the basal ganglia, the M1 is sensitive to aging, and changes in this region have been shown to correlate with impaired gait. For example, decreased M1 dopamine transport and receptor density is seen with aging [83,84], along with reduced M1 volume, which is associated with shorter stride length, longer double support time, and slower gait speed [79,85]. Further, several studies have reported M1 hypoexcitability with age, including increases in intracortical inhibition and decreases in intracortical facilitation [86,87], suggesting reductions in motor output. Interestingly, studies have shown that during the performance of motor tasks, there is an age-associated increase in the recruitment of accessory processing areas [88,89], such as the somatosensory cortex. 

### 4.3. Primary Somatosensory Cortex (S1)

The S1 a series of functions including proprioception, pain, heat, and vibration sensation, as well as tactile discrimination. Although an increase in gait variability along with a reduction in volume in brain regions such as the parietal and sensorimotor cortices has been shown with age, no changes in S1 gray matter volume have been reported. However, it is interesting to note that in S1 slice recordings from aged rats, thalamocortical activation showed increased cellular excitability, as well as increased receptive field size and suppression of responses, compared to adult animals [90,91]. Other animal studies have also shown age-associated increases in S1 neuronal excitability that may be mediated by changes in GABAergic innervation [90,91,92]. Similarly, in the clinic, a significant correlation between increased S1 excitability and impaired tactile acuity in older individuals was reported [93]. 

It is clear that aging is associated with a decline in both tactile and motor function; however, recent evidence suggests that distinct aging-sensitive mechanisms may underlie the changes reported in these brain regions [94]. Thus, future investigations targeting region-specific processes and pathways that are associated with age-dependent gait dysfunction are warranted. Furthermore, such work would also begin a thorough characterization of the different modalities that are sensitive to insulin. Recently, the administration of insulin was shown to increase the motivation to ambulate both in the clinic and in animal models [95,96,97,98], suggesting that this may be a novel, clinically relevant approach for targeting age-related gait impairments, perhaps by impacting network excitability changes in S1 (Figure 1).

## 5. The Insulin Receptor as a Potential Novel Therapeutic Target of Gait Dysregulation

### 5.1. Insulin Receptors in the Brain

The insulin receptor (IR) is widely found throughout the brain, and both IR function and expression are known to be reduced with age [99,100,101,102,103]. Interestingly, all of the regions outlined above show at least some degree of IR binding and/or mRNA expression. Autoradiographic measures obtained in rat brain slices using ^125^I-insulin showed a moderate amount of binding in the caudate putamen of the basal ganglia [104], and more recent evidence indicated that IR activity is involved with dopaminergic signalling in both the dorsal (caudate nucleus and putamen) and ventral (nucleus accumbens) striatum [105]. Further, in mice, loss of astrocytic IRs (AAV knockout) within the dorsal striatum has been shown to be associated with depressive-like behaviors as well as impaired dopamine release in brain slices [106].

Moderate insulin binding has also been detected in the frontal and parietal lobes, which include M1 and S1 [104]. These findings are supported by more recent work that reported a similar amount of IR mRNA expression within the cerebral cortex as found in the hippocampus [107], as well two other studies which showed that central insulin administration was associated with a significant increase in IR signaling in the cortex of an AD mouse model [108] and in a rat model of aging [109]. Similarly, in a mouse model of autism, the S1 was shown to be insulin-sensitive, as application of insulin to thalamocortical slices increased inhibitory post-synaptic potentials (IPSPs) while pioglitazone, an anti-diabetic drug, restored the balance between excitatory and inhibitory post-synaptic potentials [110]. Interestingly, central delivery of insulin in a mouse model of PD was associated with reduced dopaminergic cell death as well as improved performance on both the apomorphine-induced rotational test and the horizontal ladder test [111], supporting the role of IRs in the maintenance of motor function. 

### 5.2. Central Insulin Administration as a Therapeutic Approach

Intranasal insulin (INI) is a safe, reliable, and effective method of increasing IR signaling in the CNS without impacting peripheral insulin levels [112,113,114,115,116,117,118]. Animal studies indicate that INI can reduce markers of Ca^2+^ dysregulation (i.e., the afterhyperpolarization [AHP]) [119] and neuroinflammation [114,120,121,122], increase hippocampal metabolism and neuronal survival [114,122,123,124], and improve aspects of learning and memory [108,114,119,124,125,126,127,128]. Similarly, in the clinic, administration of INI is associated with enhanced memory and cognitive function in individuals with AD and mild cognitive impairment (MCI) [129,130,131,132,133], as well as cognitively normal adults [112,134,135,136]. 

**Figure 2:**
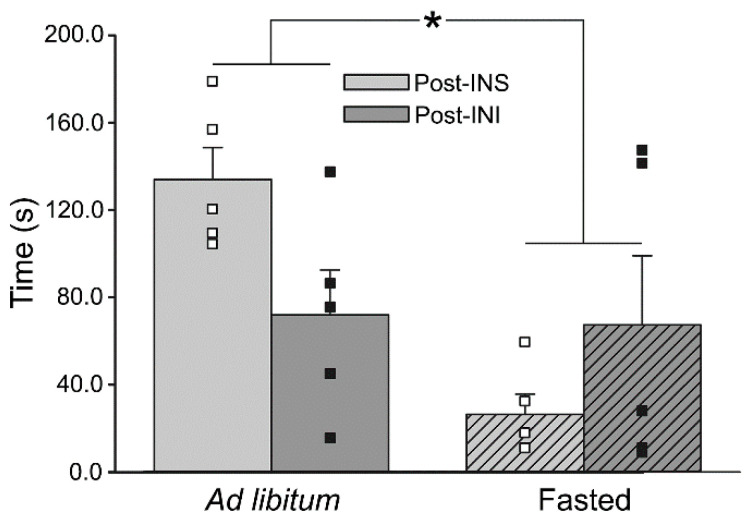
**Impact of acute INI administration and motivation on measures of ambulatory performance in aged animals.** Measures of time spent ambulating across 4 surfaces obtained in aged animals receiving INS (n = 5) or INI (n = 5). Compared to ad libitum-fed animals, a significant reduction in average time ambulating was noted in animals that were food deprived and were therefore motivated to complete the task (2-way ANOVA; F_(1,16)_ = 7.34, *p* = 0.02). Additionally, a significant interaction term was also detected (F_(1,16)_ = 6.19, *p* = 0.02), with INI-treated animals ambulating more quickly than INS-treated under ad libitum conditions, while this effect was reversed when animals were fasted. Data represent means ± SEM. Asterisk (*) indicates significance at *p* < 0.05. Figure reproduced from Lin et al., 2022 [137].

Interestingly, recent evidence suggests that central insulin administration may also be able to target other modalities associated with sensorimotor and/or motivational function. Indeed, clinical administration of INI was recently shown to increase the motivation to move [96,98], further supporting the role of IR in processes associated with ambulatory behavior. Similarly, we recently found significant interactions between INI delivery and motivation in the Fisher 344 (F344) rat model of aging (Figure 2). In this study, non-food-motivated aged animals fed an ad libitum diet took significantly less time to complete an ambulatory task following acute INI administration compared to aged, non-motivated animals treated with intranasal saline (INS). Together, these results suggest that INI may impact reward/ motivational pathways; however, despite findings of INI-mediated changes in aspects of ambulatory behavior, there is still a lack of work directly focused on investigating the role of insulin and IR activity specifically in M1 or S1. Given the importance of these regions in the control of ambulation, it is clear that investigating the impact of insulin signaling in these areas as a potential therapeutic target for the treatment of age-related gait dysregulation is needed.

While INI has been shown to impact aspects of age-dependent cognitive function and motivational status in both animal models as well as in the clinic, the exact mechanisms and pathways underlying these effects are still not clear. One potential process that has been suggested is insulin’s impact on cerebral blood flow (CBF). However, in the clinic, the role of INI in mediating CBF currently remains unclear. An early pilot study showed no impact of INI (160 IU) on CBF in the visual cortex of adult (18–34 years old), lean subjects [138], yet several others have shown significant changes in blood flow under similar conditions. For example, one group showed that acute delivery of INI (40 IU) can increase measures of CBF in the insular cortex of healthy young men (~24 years old) 35–60 min after administration [139], while another reported that acute INI significantly increased middle frontal gyrus CBF in young (~25 years old) lean participants, but decreased CBF in the hypothalamus of these same individuals [140]. Interestingly, this same study also presented evidence of increased CBF in the middle frontal gyrus, albeit only in overweight/obese subjects, arguing against the presence of central insulin resistance in overweight/obese individuals. Yet, in a more recent study, administration of 160 IU of INI decreased, rather than increased, CBF in several brain regions, including the hippocampus, insula, putamen, parahippocampal gyrus, and fusiform gyrus in overweight, young (~26 years old) males, but not in normal weight individuals [141]. Interestingly, in this study, the same level of peripheral insulin sensitivity was reported across groups, suggesting that changes in central IR activity can occur independently of changes in the periphery. In patients with T2DM, INI appears to increase perfusion in the insular cortex to a greater extent than in non-diabetic control subjects [142]. Furthermore, it also appears central insulin-sensitivity may also be preserved in aging, as another study showed that INI significantly increased perfusion in the occipital gray matter and thalamus of older individuals but not in younger subjects [143].

What is clear, is that depending on the brain region investigated and the health status or sex of the individual, insulin can have differential effects on CBF. Whether this means that brain regions have different levels of insulin-sensitivity or insulin receptor density, more research clarifying the role of insulin’s effects on CBF is needed. While current clinical evidence supports some role for insulin in the control of CBF, work measuring INI’s impact on CBF in animal models is relatively limited. Recently, we reported that chronic (9 day) administration of INI in an animal model of aging (F344 rats) was able to offset the age-dependent decrease in CBF [109]. Similarly, another study showed that in diabetic rats (STZ-treated), chronic administration of INI (2 IU/day for 14 days) significantly increased CBF compared to saline while also improving MWM performance, cholinergic function, and brain metabolism [124]. However, this study was performed only in adult rats, and thus did not investigate the impact of age. Given that some evidence has suggested that central insulin sensitivity may be preserved in the aged brain, this is an important element to consider, particularly when studying the direct impact of insulin on the brain, thus bypassing other factors that may contribute to reduced IR signaling with age.

## 6. Insulin Sensitivity in the Brain: Contradictory Evidence

At least three recognized factors may be responsible for mediating a reduction in insulin signaling in the brain, including (1) reduced receptor number, (2) reduced IR sensitivity, or (3) reduced transport of the ligand from the periphery. However, despite evidence supporting the presence of all three of these factors, most studies measuring traditional intracellular IR signaling (i.e., IRS-1, pAKT/AKT, etc.) are studying a symptom of one of the factors listed above, as it is not just reduced receptor sensitivity that can lead to diminished downstream insulin signaling, but also decreased IR density and ligand availability, albeit in different ways. Below, we examine prior work reporting reduced brain IR signaling, and also present important findings that suggest other factors (e.g., reduced ligand transport) should also be considered when discussing the development of central insulin resistance.

### 6.1. Evidence Supporting Brain Insulin Resistance in Aging and AD

The hypothesis of brain insulin resistance has historically been characterized by an overall reduction in insulin binding and downstream IR signaling, decreased brain insulin levels, and diminished insulin-mediated processes [144,145]. Prior literature strongly supports this hypothesis during normal aging as well as across numerous disease states, such as MCI, AD, and diabetes [100,146,147,148,149,150]. For example, a recent study reported that hyperinsulinemia in aging was associated with reduced IR signaling along with impaired neuronal function and metabolism [151]. Another study also reported that the AD brain shows significant reductions in several downstream IR signaling molecules, such as IRS-1 and PI3K [152]. Interestingly, however, this study also reported that insulin (ex vivo delivery) was able to bind and activate the IR in hippocampal formation slices of patients diagnosed with AD, suggesting these receptors were still sensitive to the ligand. 

Similar findings have also been reported in animal models. In a study of APP/PS1 mice, aged animals had significant elevations in serine-phosphorylated IRS-1, which is known to be associated with poor cognitive performance and impaired IR signaling, along with reduced expression of downstream IR signaling transcripts [153]. Dineley and colleagues also reported alterations in several aspects of canonical IR signaling pathways in two different animal models of amyloidosis (Tg2576 and 3xTg mice) [154]. While evidence is provided for reduced insulin signaling in some IR downstream effectors in this study, surprisingly, several molecules showed increased expression in older (16 mo) 3xTg mice compared to young, including IRS-1, pAKT, and GSK3β. Of note, both of these studies report on the presence of central insulin resistance in the absence of peripheral insulin resistance, suggesting that these two processes may be independent. Similarly, another study in aged APP/PS1 mice reported that direct delivery of insulin to the hippocampus (reverse microdialysis) significantly elevated insulin levels and IR signaling (pAKT/AKT), suggesting that the AD brain is still responsive to the ligand [155].

Finally, a study investigating central insulin resistance in clinical AD samples as well as in two different animal models of AD similarly revealed significant elevations in hippocampal pSer IRS-1, further supporting the presence of reduced insulin signaling [156]. Taken together, it is clear that aging and AD are both associated with impaired elements that are downstream from the IR. However, measures of these effectors provide only a partial characterization of insulin resistance in the brain, as they represent latter events resulting from alterations in other upstream insulin-related processes (i.e., reduced IR density, decreased ligand availability, etc.) that could occur during the development of this phenotype. Below, we present evidence that IR sensitivity may be preserved across age and AD, suggesting that other initiating events could underlie changes in downstream insulin signaling in the brain.

### 6.2. Evidence Supporting Preserved Insulin Sensitivity with Aging

The very fact that INI appears to be an effective approach to elevate central IR signaling and alter mood, appetite, and cognition across the aging spectrum, as well as in individuals with significant amyloid pathology and/or AD and MCI-associated cognitive impairment, suggests that the traditional premise of central insulin resistance as defined by the inability of the IR to respond to the ligand may not be as prevalent as previously thought. In fact, pioneering clinical work revealed significant beneficial effects of central insulin administration on declarative memory in patients diagnosed with MCI or early AD pathology [157]. More recent work mirrored these early findings, showing that INI improved both verbal word-list recall and verbal story recall in patients diagnosed with MCI/AD, albeit only those that were ApoE ε4 negative [132,158]. This suggests that while advanced amyloid pathology (ApoE ε4 positive) may be associated with reduced central insulin activity, older individuals with less pronounced neuropathological changes may still respond to elevated levels of the ligand. However, while it is well-known that aging is the most prominent risk factor for the development of AD, evidence suggests that these two processes are different from one another, implying that insulin resistance in AD may be more pronounced than that seen during aging. In fact, there is a paucity of work directly measuring the impact of insulin on aged tissue.

Recently, our lab and several others have suggested the broadening of the definition of brain insulin resistance and have even questioned its manifestation in the aging brain [100]. For example, using applications of insulin both directly (ex vivo in the slice) as well as indirectly (in vivo INI), we showed, for the first time, that the Ca^2+^-dependent AHP, a potential that can prevent neuronal firing, is sensitive to insulin [119,159]. Importantly, in these studies, the insulin-mediated reduction in this potential was greater in slices from the aged animal compared to those from the young, highlighting preserved insulin sensitivity in these animals. More recently, we also showed that administration of chronic INI (equivalent to 10 IU/day for 9 days) in F344 rats significantly increased CBF compared to intranasal saline, but only in the aged animal [109]. Early work in animal models supports the presence of preserved insulin activity during aging, as no reduction in insulin binding was detected across multiple brain regions in aged rats compared to young [101]. Further, work from another group has shown that while endogenous IR activity was reduced in the aged mouse, application of intracerebroventricular insulin was associated with a significant increase in downstream IR signaling molecules that were comparable to levels measured in the young animal [160]. This, in combination with regional-specific elevations in IR signaling (pAKT/AKT) in these same animals after acute INI administration, suggests that central insulin sensitivity may indeed be preserved during aging, provided there was adequate availability of the ligand.

One potential process that could explain reports of reduced IR activity concurrent with preserved receptor sensitivity following exogenous insulin administration is reduced levels of circulating insulin in the brain. While early investigations of IRs in the brain suggested that insulin may be locally produced, this hypothesis remains controversial [145], with the bulk of evidence strongly supporting the transport of the ligand from the periphery across the blood–brain barrier (BBB) through a saturable system [161,162]. Studies of IR binding using quantitative autoradiography suggests that the majority of insulin transport occurs at the choroid plexus, capillary beds, and the olfactory bulbs [161], coinciding with this region’s high density of IR expression [163,164]. A reduction in this transport has been suggested to underlie central insulin resistance in aging and AD [162]. Interestingly, insulin transport has also been shown to be altered by the presence of peripheral insulin resistance (T2DM), as highlighted by work in animal models showing that hyperinsulinemia is associated with reduced BBB IR density [165] and compromised BBB integrity and function [166]. Taken together, it may be that the greatest hurdle limiting central IR signaling in the aged brain is not the inability of the IR to respond to the ligand, but instead is the inability of insulin to enter the brain, as suggested by Sartorius and Heni [160,167].

### 6.3. Could INI Be a Therapeutic Approach for Gait Dysfunction?

**Figure 3:**
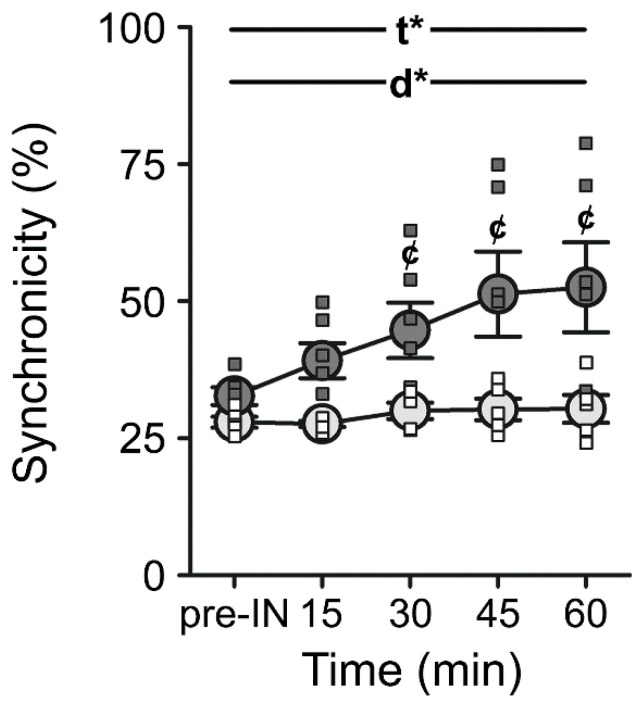
**Analysis of the S1 neuronal Ca^2+^ network in response to intranasal saline (INS) or INI in aged F344 animals.** Measures of synchronicity were derived from a correlation matrix across ~1000 neurons in each group. Main effects of both time following intranasal delivery (2-way ANOVA; F_(1.36,12.25)_ = 9.90, *p* = 0.01) and INI (F_(1,9)_ = 7.43, *p* = 0.02) were detected. Data represent means ± SEM. Asterisks indicate main effects of time (t*) or drug (d*) at *p* < 0.05. Cent symbols (¢) denote Bonferroni post hoc significance (*p* < 0.05) between INS and INI at the timepoints indicated. Figure reproduced from Lin et al., 2022 [137].

Recently, exciting results have shown that insulin may target new domains outside of those associated with learning and memory processes (i.e., the hippocampus), and may be able to modify functions such as aspects of sleep [168], appetite [135], mood [169,170], and perhaps even ambulation [95,96,97,98]. We are currently investigating the potential use of INI as a therapeutic approach to ameliorate gait dysfunction. In a series of experiments imaging S1 neuronal Ca^2+^ networks in aged F344 rats using a two-photon (2P) microscope platform during tactile activation, we were able to detect changes in network variables following acute INI delivery (Figure 3). Briefly, following AAV delivery of a Ca^2+^ sensor (AAV.CamKII.GCaMP6s.WPRE.SV40; Addgene #107790) to S1, animals were imaged during tactile stimulation (5 s, 3 Hz) of both the hind- and forepaw. Extraction of the Ca^2+^ signals was accomplished using an in-house MATLAB code in combination with a Morse continuous wavelet transform approach, allowing us to detect the power of the signal across multiple frequencies (0.06–13 Hz). Outcome measures included network synchronicity, connection lengths, and connectivity. We showed that within 15 min, INI was able to significantly increase Ca^2+^ network synchronicity in S1 neurons compared to intranasal saline (Figure 3). 

**Figure 4:**
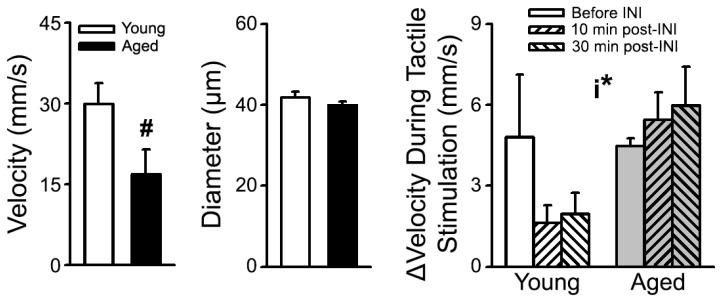
**Measures of S1 CBF at rest and in response to tactile stimulation following INI delivery.** (**Left**) Compared to young (n = 3) F344 animals, aged (n = 3) animals showed a trend (#) for reduced absolute red blood cell velocity in S1 (Student’s *t*-Test; *p* = 0.09). (**Middle**) No change in vessel diameter was detected (Student’s *t*-Test; *p* > 0.05). (**Right**) Measures of Δvelocity during tactile activation was measured across age and in response to INI administration. A significant interaction (i*) was noted, with young animals responding to INI with reduced Δvelocity while aged animals responded with an increase (2-way RM ANOVA; F_(2,17)_ = 4.43; *p* = 0.05) [173].

In the same animals, INI was also able to decrease the time taken to ambulate down a corridor during a gait behavioral task [137] perhaps indicating that the changes in ambulation were reflected by alterations in network communication. Further, in this same model, we also found that INI was able to significantly alter measures of CBF (Figure 4). Following retro-orbital injections of 5% rhodamine dextran (500 kDa), medium-sized vessels in S1 were imaged on the 2P microscope prior to and during tactile stimulation (5 s, 3 Hz). To monitor red blood cell movements across vascular beds, we used a MATLAB code to extract proxies of CBF (radon transform to extract the angle with the greatest contrast). Vessel diameter over time was derived via line scans perpendicular to the vessel axis, while red blood cell velocity was determined using repeated line scans through the lumen of the vessel [171,172]. While no major aging effect was identified on measures of change in red cell velocity (Δvelocity) during tactile activation, a significant interaction term was present (2-way repeated measure [RM] ANOVA; F_(2,7)_ = 4.43; *p* = 0.05), whereby aged animals responded with an increase in CBF following INI, as previously reported [109], while young animals responded with a decrease (Figure 4, Right). Once again, these findings further emphasize that irrespective of age, insulin is capable of altering key central processes. 

## 7. Summary and Conclusions

It is abundantly clear that one of the most direct tests for the presence of insulin resistance within a target tissue must include the application of insulin to the system, as this addresses at least two factors that may underlie the development of this pathology (i.e., decreased sensitivity or IR density). Of course, the third factor in this triad of potential pathways must also be addressed via studies focused on BBB transport of the ligand. Thus, we propose that in order to develop new testing paradigms, and better characterize the mechanisms underlying age-related insulin resistance and gait dysfunction, emerging technologies to investigate these changes must be used not only in young adult animals, but in aged animals as well.

Here, we provided an overview of underlying structural and functional changes of key brain regions associated with locomotor stability, discussed current findings associated with age-dependent insulin resistance in the brain, and also emphasized that these domains could represent novel targets of INI. These topics are particularly relevant, given that falls are a leading cause of injuries in older individuals, that aging is associated with central insulin resistance, and that INI is a safe and effective approach to increase IR signalling. Further, we also presented new results using 2P imaging approaches to address neuronal Ca^2+^ network communication and neurovascular unit coupling in response to both age and insulin. Ongoing studies in our lab are currently investigating S1 Ca^2+^ networks in head-restrained, ambulating mice to test the hypothesis that INI can offset gait impairments and increase ambulatory motivation by enhancing network synchronicity. We believe this work will help to broaden the definition of insulin resistance in the brain by asking whether this process is mediated by reduced IR sensitivity, decreased receptor density, and/or impaired BBB insulin transport.

## Data Availability

Not applicable.
